# Sight of parasitoid wasps accelerates sexual behavior and upregulates a micropeptide gene in *Drosophila*

**DOI:** 10.1038/s41467-021-22712-0

**Published:** 2021-04-27

**Authors:** Shimaa A. M. Ebrahim, Gaëlle J. S. Talross, John R. Carlson

**Affiliations:** Department of Molecular, Cellular and Developmental Biology, Yale University, New Haven, CT USA

**Keywords:** Behavioural ecology, Coevolution, Cellular neuroscience, Neural circuits

## Abstract

Parasitoid wasps inflict widespread death upon the insect world. Hundreds of thousands of parasitoid wasp species kill a vast range of insect species. Insects have evolved defensive responses to the threat of wasps, some cellular and some behavioral. Here we find an unexpected response of adult *Drosophila* to the presence of certain parasitoid wasps: accelerated mating behavior. Flies exposed to certain wasp species begin mating more quickly. The effect is mediated via changes in the behavior of the female fly and depends on visual perception. The sight of wasps induces the dramatic upregulation in the fly nervous system of a gene that encodes a 41-amino acid micropeptide. Mutational analysis reveals that the gene is essential to the behavioral response of the fly. Our work provides a foundation for further exploration of how the activation of visual circuits by the sight of a wasp alters both sexual behavior and gene expression.

## Abstract (web summary)

Parasitoids exploit host bodies for reproduction, selecting for host defences. A new host defence is reported, in which adult *Drosophila* accelerate mating behaviour at the sight of certain parasitoid wasps, mediated by the upregulation of a nervous system gene that encodes a 41-amino acid micropeptide.

## Introduction

*Drosophila* in the wild suffers massive mortality from the attacks of parasitoid wasps. As many as 80% of *Drosophila* larvae in natural environments may be killed by wasps that lay eggs in them^1^. These eggs hatch into larval wasps that devour the fly larva from within^2^. Adult wasps then emerge and mate, after which female wasps search for new hosts in which to begin the cycle anew^3^.

As many as 350,000 species of parasitoid wasps may inhabit the natural world^4^, an indication of their enormous importance in ecology and evolution. The species they parasitize have evolved diverse defenses to protect themselves against these parasitoids. Some defenses operate at the cellular level. Attacked larvae, including those of *Drosophila*, may encapsulate the wasp egg with hemocytes and melanin^5^. Other larval defenses act at the behavioral level. *Drosophila* larvae show a remarkable rolling response at the onset of a wasp attack^6,7^. The larva rolls toward the wasp, winding its ovipositor around the larval body. This rolling often flips the wasp off balance and ends the attack.

Adult *Drosophila* behavior is also affected by exposure to wasps. Female flies reduce their rate of oviposition, which may allow the female to lay more eggs later at another oviposition site where the threat of wasp attack is lower^8,9^. Females exposed to wasps also prefer to lay eggs in food sources that contain alcohol, to which developing wasps may be more sensitive than developing flies^10^.

These diverse defensive behaviors are driven by different kinds of neurons. Rolling behavior of larvae is mediated by nociceptive neurons^6^, and the reduction in oviposition can be driven by visual cues^8,10^. A dedicated olfactory circuit detects odors of parasitic wasps and drives aversion responses. In larvae, olfactory receptor Or49a detects the wasp odor iridomyrmecin and drives an avoidance response^11^. In adult flies, Or49a is coexpressed with Or85f, a receptor that detects the wasp odors actinidine and nepetalactol in a class of antennal neurons called ab10B^11^. These neurons drive avoidance of food substrates and oviposition sites that contain wasp odors.

Along with the search for food sources and oviposition sites, mating is one of the most robust and essential of *Drosophila* behaviors. The mating behavior of fruit flies has been studied for over 100 years^12^. Males and females exchange a variety of cues, and successful mating depends on the execution of stereotyped male behaviors and on female receptivity^13^. Given the high frequency at which flies and their parasitoids encounter each other in nature, we wondered whether flies had evolved a mechanism that would allow mating behavior to respond adaptively to the presence of parasitoids.

Here we find that exposure to parasitoid wasps affects sexual behavior between male and female flies: surprisingly, it is accelerated. Flies begin to copulate more quickly. The effect is observed in five different species of *Drosophila*, and can be induced by several species of parasitoid wasps that parasitize *Drosophila*, but not by species that do not. The effect depends on visual cues, is eliminated by a mutation ablating photoreceptor function, and is impaired in a fly in which LC4 visual projection neurons (VPNs) are blocked. Sexual behavior is affected after a female fly has been in visual contact with a wasp in a different compartment. This visual contact induces expression of a gene encoding a micropeptide of 41 amino acids in the *Drosophila* nervous system. Mutational analysis shows the gene is required for the mating acceleration. These results implicate a micropeptide in a novel behavioral response to a threat that is widespread in the insect world.

## Results

**Fig. 1:**
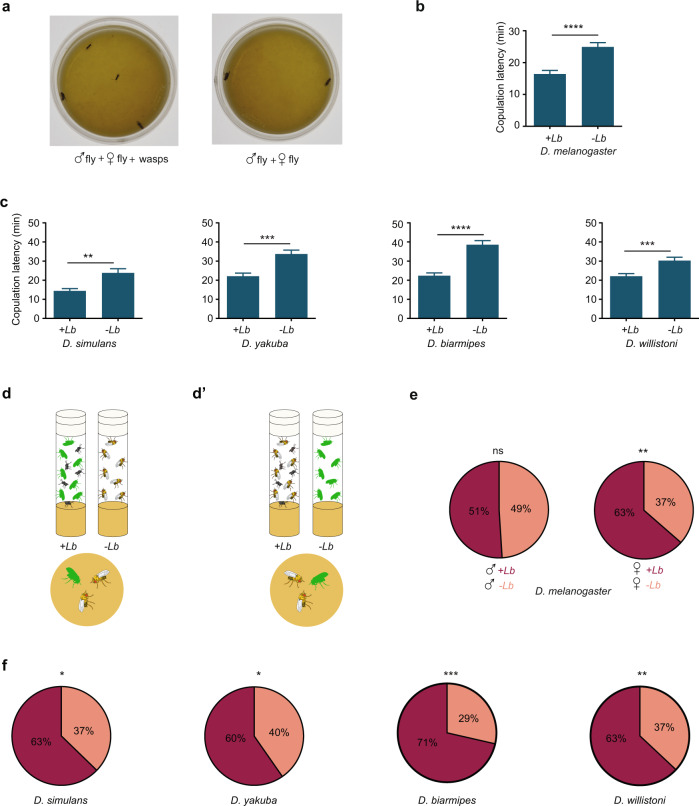
Exposure of *Drosophila* to wasps accelerates sexual behavior. **a** Courtship arena containing a male and virgin female fly with (left) and without (right) two wasps, one male and one female. **b** Copulation latency of *D. melanogaster*. *p* < 0.0001, two-tailed Mann–Whitney test, *n* = 105 for +Lb, i.e., exposed to wasps, *n* = 102 for unexposed, error bars = SEM. The experiment was allowed to run for 1 h. **c** Copulation latency of other *Drosophila* species while in arena. Four wasps, two males and two females, were used for exposure. Two-tailed Mann–Whitney test. *D. simulans*, *p* = 0.0035, *n* = 67 (exposed), *n* = 63 (unexposed); *D. yakuba*, *p* = 0.0001, *n* = 62, 63; *D. biarmipes*, *p* < 0.0001, *n* = 62, 62; *D. willistoni*, *p* = 0.0009, *n* = 93, 92. Error bars = SEM. **d** Competition paradigm. In the left vial, fluorescently labeled flies (green) are incubated with wasps; in the right vial, unlabeled flies are incubated without wasps; in the arena below, a labeled exposed fly and an unlabeled unexposed fly of the same sex are allowed to compete for a fly of the opposite sex. **d**′ Competition paradigm. In the left vial, unlabeled flies are incubated with wasps; in the right vial, labeled flies are incubated without wasps; in the arena below, an unlabeled exposed fly and a labeled unexposed fly of the same sex are allowed to compete for a fly of the opposite sex. **e** Left, competition between a male exposed to *L. boulardi* and an unexposed male for an unexposed female; dark shading indicates the percentage of competitions won by the exposed male (*p* = 0.843, chi-squared test, *n* = 102). Right, a female exposed to *L. boulardi* and an unexposed female are placed in an arena with an unexposed male; dark shading indicates the percentage of pairings in which the exposed female copulated (*p* = 0.0038, chi-squared test, *n* = 115). **f** Experiments comparing the mating success of an exposed and unexposed female of other species, placed with a male of the same species. Chi-squared test. *D. simulans* (*p* = 0.0129, *n* = 102); *D. yakuba* (*p* = 0.0350, *n* = 119); *D. biarmipes* (*p* = 0.0001, *n* = 84); *D. willistoni* (*p* = 0.0057, *n* = 119).

We asked whether the mating of male and female fruit flies would be affected by the presence of parasitoid wasps. We placed a pair of *D. melanogaster* flies in a small Petri dish, either with or without parasitoid wasps (Fig. 1a). In an initial experiment we used the wasp *Leptopilina boulardi*, which specializes on *D. melanogaster* and on closely related fly species^14^.

We expected that the presence of the parasitoids, a lethal threat to reproductive success, would delay mating. We were surprised to find exactly the opposite. In the presence of the wasps, the mean time to copulation was 16 min, compared to 25 min in the absence of the wasp, i.e. a 36% reduction in copulation latency (Fig. 1b, ****p < 0.0001, two-tailed Mann–Whitney test, *n* = 105, 102). The exposure to wasps did not reduce the fraction of pairs that copulated in this experiment: copulation occurred in 80% of pairs that were not exposed to wasps and in 86% that were exposed *(p* > 0.05, chi-squared test). We then tested four additional *Drosophila* species closely related to *D. melanogaster* and found the same acceleration to copulation in all four (Fig. 1c).

Exposure to wasps could affect the behavior of either the male fly or the female fly. We tested these possibilities through competition experiments. If exposure increased male motivation, we might expect an exposed male to outcompete an unexposed male when both males were placed in a chamber with a single unexposed female. We placed fluorescently labeled male flies in a chamber with wasps for 2 h, and in parallel placed unlabeled male flies in a separate chamber without wasps (Fig. 1d). We then allowed a fluorescent exposed male to compete with an unexposed male for the same female in a small arena. To control for any effect of the fluorescent label, we also performed the reciprocal experiment, i.e. we exposed unlabeled males to wasps and allowed them to compete with unexposed, labeled males (Fig. 1d′). The results of the two reciprocal experiments were scored blind and averaged. We found that exposed males and unexposed males were equally successful in mating competitions (Fig. 1e, left).

By contrast, when we placed an exposed female and an unexposed female together with an unexposed male, in 63% of tests the exposed female was the one that copulated (Fig. 1e, right; **p < 0.01, chi-squared test, *n* = 102, 115). These results suggested the hypothesis that exposure of females to wasps increases their receptivity to males. We tested this hypothesis by pre-exposing females of the other four *Drosophila* species to wasps. In all four cases, pre-exposed females were more likely to mate than non-exposed females (Fig. 1f).

**Fig. 2:**
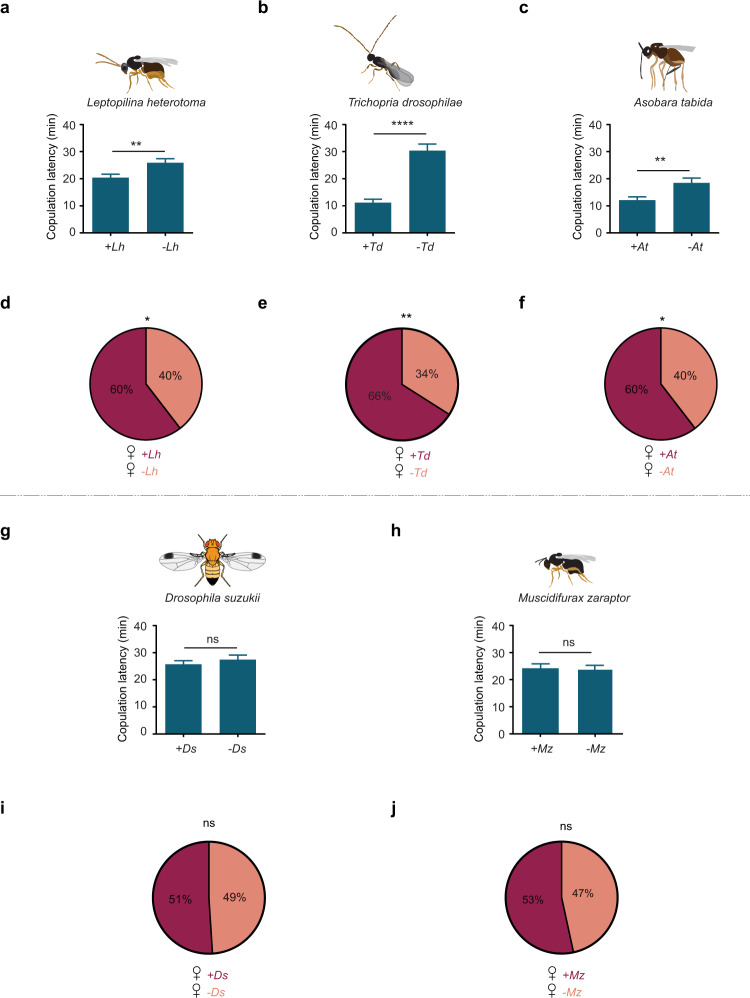
Effect of exposure to other insect species on sexual behavior of *D. melanogaster*. **a** *Leptopilina heterotoma*, *n* = 121 (exposed), 100 (unexposed). *p* = 0.0044, two-tailed Mann–Whitney test. Error bars = SEM. **b** *Trichopria drosophilae*, *n* = 67 (exposed), 67 (unexposed). *p* < 0.0001. **c** *Asobara tabida*, *n* = 81 (exposed), 85 (unexposed), *p* = 0.0051. **g** *Drosophila suzukii*, *n* = 121 (exposed), 119 (unexposed), *p* = 0.9592. **h** *Muscidiflurax zaraptor*, *n* = 101 (exposed), 101 (unexposed), *p* = 0.9568. Error bars in **b**, **c**, **g**, **h** are SEM. The statistical test used in **b**, **c**, **g**, **h** is a two-tailed Mann–Whitney test. In each case a male *D. melanogaster* and a virgin female *D. melanogaster* were placed in an arena with one male and one female of the indicated species. **d**–**f**, **i**, **j** A female *D. melanogaster* exposed to the indicated species and an unexposed female *D. melanogaster* were both placed in an arena with a male *D. melanogaster* as in Fig. 1d, d′. Dark shading indicates the percentage of cases in which the exposed female mated. **d** *L. heterotoma* *p* = 0.0352, chi-squared test, *n* = 100. **e** *T. drosophilae* *p* = 0.0018, *n* = 100. **f** *A. tabida* *p* = 0.0464, *n* = 91. **i** *D. suzukii* *p* = 0.9196, *n* = 98. **j** *M. zaraptor* *p* = 0.5546, *n* = 103. The statistical test used in **e**, **f**, **i**, **j** is a chi-squared test.

We next asked whether exposure to other species of parasitoid wasps also accelerated mating behavior. We first tested *Leptopilina heterotoma*, which is closely related to *L. boulardi*, and subsequently *Trichopria drosophilae* and *Asobara tabida*; all of these species have been shown previously to attack *Drosophila melanogaster* larvae or pupae^15^. When wasps of any of these three species were placed in an arena with a pair of *D. melanogaster* flies, copulation began sooner (Fig. 2a–c). As a further test, a female fly that was pre-exposed to each wasp species was more likely to copulate than a non-exposed female fly when both were placed in an arena with a male (Fig. 2d–f).

Does the presence of any other insects whatsoever accelerate *Drosophila* mating? The presence of another species of *Drosophila, D. suzukii*^16^, did not have any of these effects on the mating of a pair of *D. melanogaster*, and neither did the presence of *Muscidiflurax zaraptor*^17^, a parasitoid that deposits eggs in larger flies (Fig. 2g–j).

**Fig. 3:**
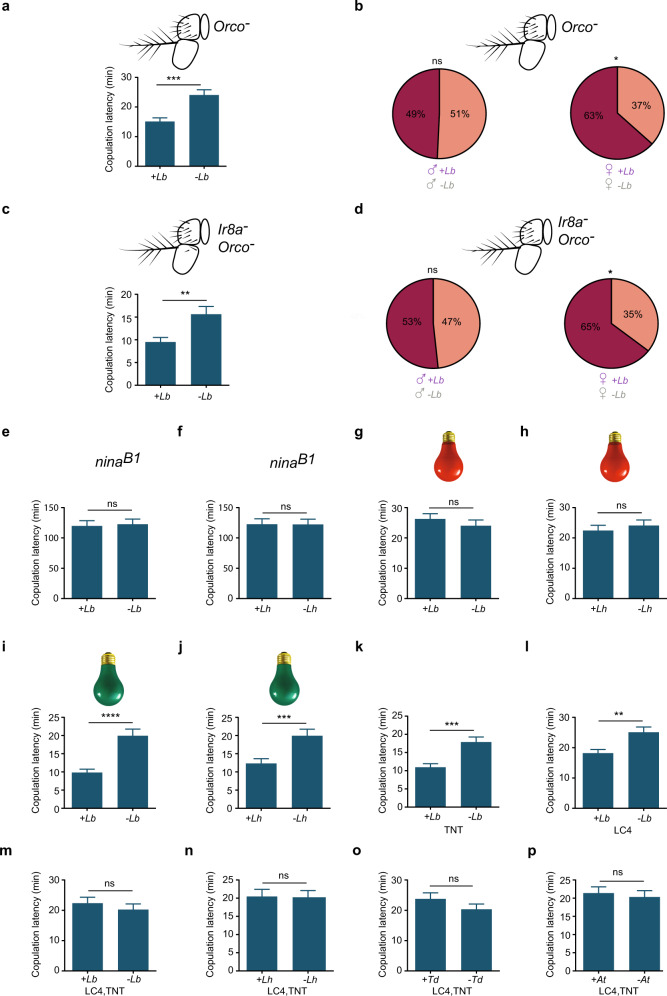
Mating acceleration depends on visual cues. **a** Mating of an *Orco*^*1*^ mutant is affected by exposure to *L. boulardi*. *p* = 0.0001, two-tailed Mann–Whitney test, *n* = 79 (exposed), 76 (unexposed). Error bars = SEM. **b** Left, competition experiments between an exposed *Orco*^*1*^ male and an unexposed *Orco*^*1*^ male, for an *Orco*^*1*^ female (*p* = 0.9042, chi-squared test, *n* = 69). Right, an exposed *Orco*^*1*^ female and an unexposed *Orco*^*1*^ female are placed in an arena with an *Orco*^*1*^ male (*p* = 0.0389, chi-squared test, *n* = 60). Dark shading indicates the percentage of cases in which the exposed animal mated. **c** An *Ir8a; Orco*^*1*^ double mutant is affected by exposure to *L. boulardi*. *n* = 66 (exposed), 67 (unexposed), *p* = 0.0085. **d** Left, competition experiments between an exposed and unexposed *IR8a; Orco*^*1*^ male, for an *IR8a; Orco*^*1*^ female (*p* = 0.8974, chi-squared test, *n* = 60). Right, an exposed and unexposed *IR8a; Orco*^*1*^ female are placed in an arena with an *IR8a; Orco*^*1*^ male (*p* = 0.0223, chi-squared test, *n* = 62). **e** A *nina*^*B1*^ mutant is not affected by exposure to *L. boulardi* under room light, *n* = 67 (exposed), 66 (unexposed), *p* = 0.7904. We note that this mutant is slow to mate. **f** *nina*^*B1*^ is not affected by exposure to *L. heterotoma* under room light, *n* = 69 (exposed), 68 (unexposed), *p* = 0.9598. 2 male and 2 female wasps were used in **e**–**j**. **g** Our Canton-S wild type strain is not affected by exposure to *L. boulardi* under dim red light *n* = 74 (exposed), 74 (unexposed), *p* = 0.195. **h** Canton-S is not affected by exposure to *L. heterotoma* under dim red light *n* = 74 (exposed), 74 (unexposed), *p* = 0.6537. **i** Canton-S is affected by exposure to *L. boulardi* under green light, *n* = 68 (exposed), 68 (unexposed), *p* < 0.0001. **j** Our Canton**-**S wild-type strain is affected by exposure to *L. heterotoma* under green light, *n* = 64 (exposed), *n* = 65 (unexposed), *p* = 0.0005. **k** Exposure to *L. boulardi* affects copulation latency of parental control TNT: *n* = 63 (exposed), 63 (unexposed), *p* = 0.0001. **l** Exposure to *L. boulardi* affects copulation latency of parental control LC4: *n* = 82 (exposed), 79 (unexposed), *p* = 0.0076. **m**–**p** Flies in which LC4 neurons are blocked with TNT are not affected by exposure to the indicated four species of wasps: in **m** (*L. boulardi*), *n* = 84 (exposed), 78 (unexposed), *p* = 0.6211; in ***n*** (*L. heterotoma*), *n* = 80 (exposed), 78 (unexposed), *p* = 0.6289; in **o** (*Trichopria drosophilae*), *n* = 90 (exposed), 81 (unexposed), *p* = 0.4496; in **p** (*Asobara tabida)*, *n* = 85 (exposed), 81 (unexposed), *p* = 0.643. Error bars in **c**, **e**–**p** are SEM. The statistical test used in **c**, **e**–**p** is a two-tailed Mann–Whitney test.

We hypothesized that the wasps that affected mating produced an odor that was not emitted by the other tested species, and that this odor activated a circuit that accelerated sexual behavior. Such an odor might be detected by Or49a, Or85f, or perhaps another receptor of the Or family^18^. If an Or were essential to the acceleration in copulation onset, then the copulation latency of flies mutant for the obligate Or co-receptor Orco^19^ would be expected to be unaffected by exposure to wasps. However, we found that *Orco* mutant flies showed an acceleration in mating onset comparable to that of control flies following exposure to *L. boulardi;* the copulation latency was reduced by 38% (Fig. 3a; compare to Fig. 1b). Moreover, competition experiments again provided evidence of an effect on females but not males, as in control flies (Fig. 3b). Similar results were obtained when *L. heterotoma* was used as the wasp (Supplementary Fig. 1a, b).

Could the effect be mediated by an odor that activates an ionotropic receptor (IR) odor receptor^20^? We tested flies doubly mutant for the Orco and IR8a co-receptors; IR8a is essential for many IR-mediated responses^21^. These double mutants again showed the same mating acceleration phenotypes, for both *L. boulardi* and *L. heterotoma*, and again pre-exposure of females but not males had an effect in competition experiments (Fig. 3c, d and Supplementary Fig. 1c, d). Taken together, these results suggested that olfactory cues do not drive the acceleration of mating.

We next tested the hypothesis that the mating effect depended on visual cues. Consistent with this hypothesis, we found that a visually impaired mutant that is defective in the synthesis of visual pigments, *ninaB*^*1*^ (ref. ^22^), showed equivalent copulation latency in the presence and absence of *L. boulardi* (Fig. 3e); the same result was found with *L. heterotoma* (Fig. 3f). Likewise, when the assay was conducted in dim red light, to which *Drosophila* has very low sensitivity, copulation latency of wild type flies was not affected by the presence of either species of wasps (Fig. 3g, h). As a control, we conducted the assay in green light (~500–565 nm), to which sensitivity is much higher, and we observed effects on mating (Fig. 3i, j). The simplest interpretation of these results is that copulation dynamics is affected by visual cues emanating from wasps. Color cues are unlikely to be essential, however, since they should be minimal under green light.

To test further the hypothesis that the effect of wasps on fly mating behavior is mediated by vision, we asked whether blockage of certain VPNs affected mating behavior. VPNs transmit information from the primary visual center of the brain, the optic lobe, to higher brain regions. The most numerous VPNs in the lobula are lobular columnar (LC) neurons, which fall into multiple types based on their anatomy^23,24^. Activation of different LC types with split-GAL4 lines evokes different behaviors^25^. Silencing of one class of LC neurons, LC4, has previously been shown to reduce the escape response of *Drosophila* to predators^26,27^.

We used split-GAL4 lines to block LC4, and found that this blockage eliminated the effect of wasps on copulation latency. Parental control flies expressing a *UAS-TNT* transgene alone (“TNT”, *Tetanus Toxin Light Chain*, which cleaves synaptobrevin to block synaptic transmission), or split-GAL4 constructs alone (“LC4”), showed the expected reduction in copulation latency when exposed to *L. boulardi* (Fig. 3k, l). However, flies in which LC4 neurons were blocked (“LC4, TNT”) did not show a reduction in copulation latency, when exposed to *L. boulardi* or any of three other tested species, *L. heterotoma, Trichopria drosophilae* or *Asobara tabida* (Fig. 3m–p).

**Fig. 4:**
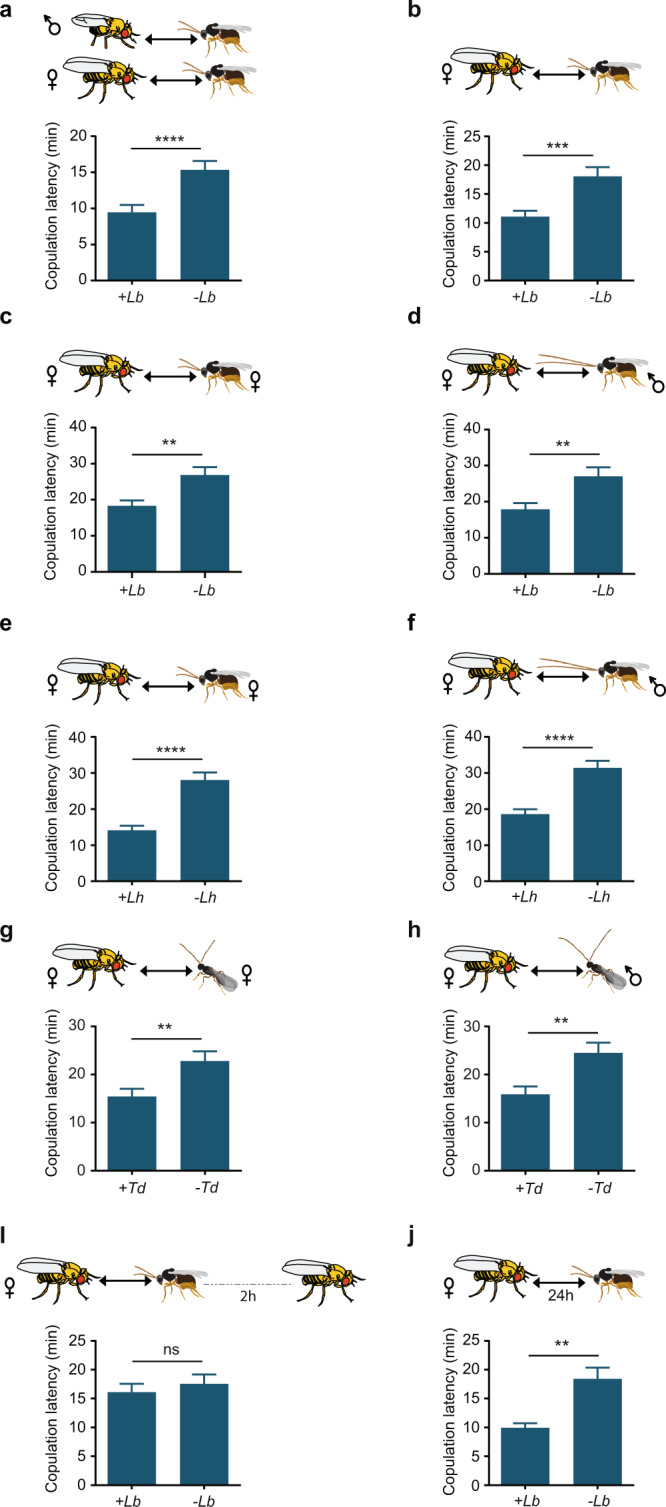
Sight of a wasp in another chamber affects sexual behavior of a fly. **a** Copulation latency of a male fly and a female fly that were each exposed separately to the sight of *L. boulardi* wasps for 2 h and then allowed to mate (+Lb, *n* = 83), compared to mating of an unexposed male and an unexposed female (−Lb, *n* = 82). *p* < 0.0001, two-tailed Mann–Whitney test. Error bars = SEM. **b** Mating of a female fly that was exposed to wasps and an unexposed male (+Lb, *n* = 71) compared to mating of unexposed flies (−Lb, *n* = 69), *p* = 0.0003. **c** Mating of a female fly that was exposed to female *L. boulardi* wasps exclusively, and an unexposed male fly (+Lb, *n* = 67) compared to mating of unexposed flies (−Lb, *n* = 67), *p* = 0.0094. **d** Mating of a female fly that was in visual contact with male *L. boulardi* wasps exclusively, and an unexposed male fly (+Lb, *n* = 63) compared to mating of unexposed flies (−Lb, *n* = 63), *p* = 0.0017. **e** Mating of a female fly that was in visual contact with female *L. heterotoma* wasps exclusively, and an unexposed male fly (+Lh, *n* = 65) compared to mating of unexposed flies (−Lh, *n* = 63), *p* < 0.0001. **f** Mating of a female fly that was exposed to male *L. heterotoma* wasps exclusively, and an unexposed male fly (+Lh *n* = 63) compared to mating of unexposed flies (−Lh, *n* = 62), *p* < 0.0001. **g** Mating of a female fly that was in visual contact with female *T. drosophilae* wasps exclusively, and an unexposed male fly (+Td, *n* = 63) compared to mating of unexposed flies (−Td, *n* = 62), *p* = 0.0058. **h** Mating of a female fly that was in visual contact with male *T. drosophilae* wasps exclusively, and an unexposed male fly (+Td, *n* = 67) compared to mating of unexposed flies (−Td, *n* = 65), *p* = 0.0014. **i** Mating of a female fly that had been in visual contact with wasps (male and female) for 2 h and then isolated for 2 h before pairing with a male fly (*n* = 69) compared with mating of an unexposed female (*n* = 69), *p* = 0.7669. **j** Mating of a female that had been in visual contact with wasps for 24 h and allowed to mate with an unexposed male (*n* = 68) compared with mating of an unexposed female (*n* = 68), *p* = 0.0038. Error bars in **b**–**j** are SEM. The statistical test used in **b**–**j** is a two-tailed Mann–Whitney test.

Visual cues were found in an earlier study to drive the depression in oviposition that follows exposure of flies to wasps^28^. In that study, visual cues from wasps in one transparent chamber were shown to affect the behavior of flies in an adjacent chamber. We found that cues from wasps could also affect sexual behavior of flies that were in visual contact with them from another chamber; copulation latency was accelerated (Fig. 4a). In this initial experiment male flies and female flies were exposed to wasps separately and then allowed to mate. We also found that female flies exposed in this manner showed accelerated copulation latency when allowed to mate with unexposed male flies (Fig. 4b), consistent with our earlier findings that wasps affected female behavior (Fig. 1e, f).

Since only female wasps are a threat to flies, we hypothesized that only female wasps would affect the mating behavior of a female fly. Female *L. boulardi* have much shorter antennae than males. However, we found that visual contact with either female or male *L. boulardi* wasps affected copulation latency (Fig. 4c, d). We also tested two other wasp species, *L. heterotoma* and *Trichopria drosophilae*, in which the antennae are also shorter in females than males, and found the same results (Fig. 4e–h).

Is the effect on the female flies permanent? We allowed female flies to be in visual contact with wasps for 2 h, and then kept them apart from wasps for 2 h before testing them with males. We found that the effect was not permanent: there was no difference between the mating behavior of exposed female flies that had been away from wasps for 2 h and unexposed female flies (Fig. 4i).

We hypothesized that if we increased the time of exposure by an order of magnitude, that female flies would adapt to the cues and their mating would no longer be affected. However, we found that if female flies were in visual contact with *L. boulardi* for 24 h and then tested, mating was accelerated. In fact, the copulation latency was reduced severely, by 46% (Fig. 4j).

**Fig. 5:**
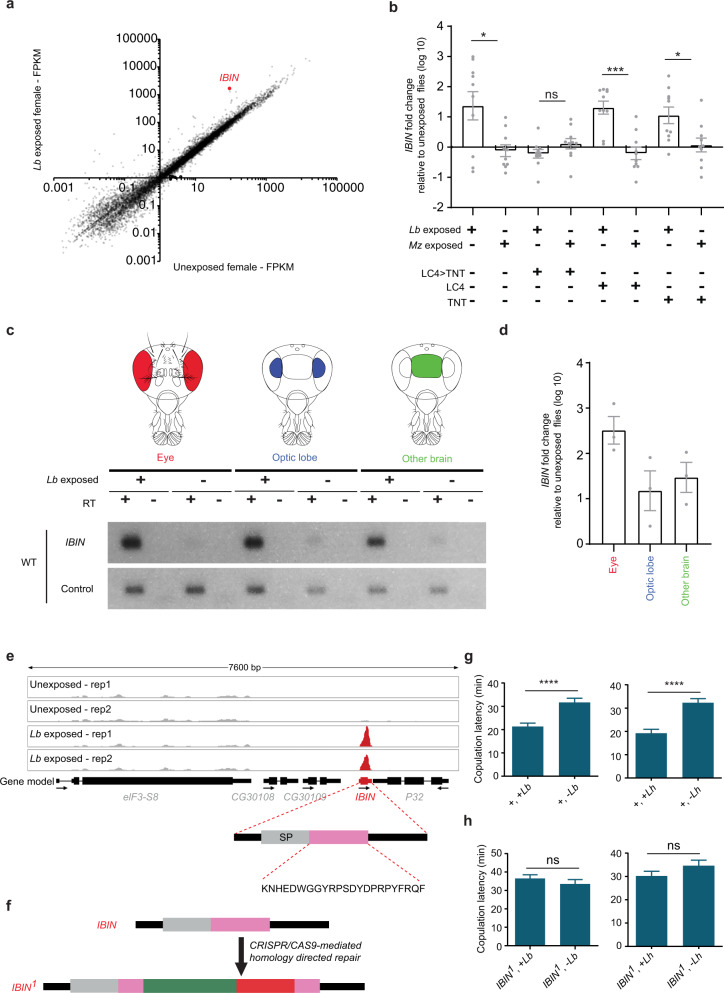
Exposure to wasps affects gene expression. **a** Head transcriptomes of flies that have been in visual contact with *L. boulardi* for 2 h, and of control flies. The transcriptome is based on two biological replicates. FPKM fragments per kilobase of transcript per million mapped reads. **b** RT-qPCR analysis of *IBIN*. Exposure to *L. boulardi* or *M. zaraptor* was for 2 h. *p* = 0.046, 0.07, 0.0008, 0.019 (from left to right), **p < 0.01, Mann–Whitney *U* test, two-tailed, *n* = 10. Error bars = SEM. **c** RT-PCR amplification from the indicated tissues. The eye tissue includes both retina and lamina. RT reverse transcriptase. The control gene is *eIF3c*. RT(−) refers to a negative control where no reverse transcriptase is added to ensure that genomic DNA is not being amplified. *n* = 2. **d** RT-qPCR analysis of *IBIN* from the indicated dissected tissues of flies that had been in visual contact with *L. boulardi* for 2 hv. from unexposed control flies. *n* = 3. Error bars = SEM. **e** RNA-seq reads (*y*-axis = 0–8000 reads), *n* = 2. SignalP (version 5.0) was used to predict the presence of signaling peptide (SP in figure) and the cleavage site, and DeepLoc (version 1.0) was used to predict its extracellular localization. **f** Generation of a mutant *IBIN*^*1*^ allele. Green indicates core promoter GAL4 sequences; red indicates 3XP 3-DsRed sequences. **g**, **h** Copulation latency of *IBIN*^*1*^ mutant flies and *wCS* control flies that had been paired with wasps (*L. boulardi)*. Two-tailed Mann–Whitney test, *n* = 67 in all cases. Error bars = SEM. In **g**, *p* < 0.0001 (left and right). In **h**, *p* = 0.377 (left), *p* = 0.1718 (right).

We wondered if gene expression was affected by exposure to wasps in this paradigm. As an initial screen, we carried out a differential RNA-seq analysis of the heads of female flies that had been exposed to *L. boulardi* for 2 h and heads of unexposed females. We identified 10 genes whose expression level was increased significantly (adjusted *p* value < 0.05) in each of two differential expression platforms (Fig. 5a and Supplementary Fig. 2). Among these 10 genes, the one that showed the greatest increase was *IBIN (Induced by Infection*, a gene that encodes a micropeptide*)*; its expression increased by ~20× (the log_2_-fold change was 4.2 by CuffDiff and 4.6 by DESeq2). IBIN was not associated with a gene ontology (GO) term, but the other nine genes are associated with the GO terms “immune system” and/or “response to stress.” Six of these other genes encode canonical antimicrobial peptides. No gene showed a decrease in expression level by our statistical criteria.

We attempted to confirm and extend this result with quantitative real-time reverse transcriptase PCR (RT-qPCR). We again found an increase of ~20× in the expression of *IBIN* in the heads of females exposed for 2 h to *L. boulardi* relative to heads of unexposed females (Fig. 5b, left bar). Exposure to the parasitoid wasp *M. zaraptor* did not increase the level of *IBIN* (Fig. 5b), just as exposure to this wasp did not affect mating behavior (Fig. 2h, j).

Blockage of LC4 neurons with *UAS-TNT* (“LC4 > TNT”) also eliminated the increase of *IBIN* induced by exposure to *L. boulardi* (Fig. 5b), consistent with its inhibition of the behavioral effect (Fig. 3m). As expected, parental control lines carrying *UAS-TNT* or *split-GAL4* constructs alone showed increases following *L. boulardi* exposure, but not *M. zaraptor* exposure (Fig. 5b).

We confirmed these results in an independent experiment with different sources of tissue and a different method. We dissected eyes, optic lobes, and brain tissue from which optic lobes had been removed, and performed RT-PCR. An *IBIN* amplification product was easily visible in all three tissue preparations in flies that had been exposed to *L. boulardi* (Fig. 5c and Supplementary Fig. 3). The amplification product appeared upregulated compared to unexposed tissues in this initial PCR experiment. Upregulation of *IBIN* was not observed in LC4 > TNT flies (Supplementary Fig. 3). We then confirmed by RT-qPCR that *IBIN* was upregulated in all three of these tissues (Fig. 5d; note log scale). The simplest interpretation of these results is that the micropeptide is upregulated in nervous tissue following exposure to *L. boulardi*.

Remarkably, *IBIN* was recently shown to be induced in second-instar *Drosophila* larvae 48 h after infection by *L. boulardi*^29^. *IBIN* was also the gene most highly induced by infection of *Drosophila* adults with the bacterium *Micrococcus luteus*^29^. The gene is induced in immune-responsive tissues, and it can be activated by either the Toll or immune deficiency (Imd) pathways, which is unusual. Its overexpression leads to increased survival after bacterial infection, although the mechanism is unclear. Adult overexpression of *IBIN* also leads to elevation of sugar levels in the hemolymph, and of Hsp70Bb, a stress-responsive gene.

An environmental stressor was also recently found to upregulate *IBIN* in males^29^. Four days of social isolation, a stressor that modulates behavior widely across animal phylogeny, upregulated *IBIN* in male heads.

The *IBIN* gene was originally annotated as a long non-coding RNA and referred to as CR44404 or *lincRNA-IBIN*. However, it contains an open reading frame that is predicted to encode a micropeptide of 41 amino acids (Fig. 5e). This micropeptide is conserved among a variety of *Drosophila* species (Supplementary Fig. 4). It is predicted to have a signal peptide that is cleaved so as to generate an extracellular micropeptide of ~23 amino acids.

To test directly whether *IBIN* is required for the effect of wasp exposure on fly mating behavior, we generated a deletion mutation of *IBIN* by CRISPR/Cas9 genome editing (Fig. 5f). We backcrossed the *IBIN* deletion allele for five generations against our control line to minimize the possibility of genetic background effects. We found that while the control line shows the expected acceleration in copulation latency with *L. boulardi* and *L. heterotoma*, the *IBIN* mutant did not (Fig. 5g, h). The simplest interpretation of these results is that the micropeptide is required for the acceleration of mating behavior.

## Discussion

We have found that the mating behavior of *Drosophila* accelerates following exposure to a parasitoid wasp. Exposed flies start mating more quickly. This acceleration effect is conserved among five *Drosophila* species tested, and the effect is elicited by exposure to several species of wasps that parasitize *Drosophila*, but not by other species that do not.

We were surprised by the positive valence of the effect, i.e. an acceleration. In attempting to interpret this effect, we considered that parasitoid wasps are not a direct threat to adult flies, but rather to their offspring. Accordingly, if an opportunity to mate arises in the presence of a wasp, we speculate that it could still be beneficial to mate, but to complete the process expeditiously with a reduced investment of energy and time so as to conserve resources for the next, crucial step: the search for an oviposition site elsewhere with lower danger of wasp attack^30^.

The effect is mediated by exposure of the female fly, as shown by the comparison of mating success between exposed and unexposed females (Fig. 1e) and the finding that latency is shortened when females alone are exposed to wasps (e.g. Fig. 4b–g). Perhaps females, which have greater parental investment than males in their offspring, have greater incentive to execute a mating strategy that improves the survival chances of their offspring. Thus the female fly would take advantage of the opportunity to mate, but would be influenced by the need to search for another food source for its offspring, a source where wasps are not present and a male fly may or may not be present. We note that both theory and experimental data support the existence of mechanisms by which exposure to parasitoid wasps may exert a rapid effect on aspects of fly reproduction (e.g. production of recombinant offspring^31^); it is conceivable that the acceleration of mating we have observed is an aspect of such a mechanism.

In interpreting this behavioral effect, it is important to appreciate the massive lethality imposed on *Drosophila* by parasitoid wasps in nature^1^. In an environment where 80% of larvae are killed by wasps, behavioral changes may be selected for strongly.

The mating effects are mediated by vision, not olfaction, as demonstrated by their dependence on light, on a critical photoreceptor gene, and on visual system projection neurons that have previously been implicated in escape behavior^26,27^; the role of vision is also consistent with the finding that fly mating is affected by visual contact with wasps held in a different chamber. The visual system also mediates the effects of wasp exposure on oviposition^10^, although the olfactory system mediates certain aversion responses in other paradigms^11^. The mating acceleration is elicited by exposure to several wasp species that parasitize *Drosophila*, but not by other species, inviting future investigation into the precise nature of the visual cues that drive this response.

Visual contact with wasps is accompanied by changes in gene expression. A gene whose expression increases dramatically, *IBIN*, encodes a micropeptide of 41 amino acids. The increase in expression of this gene is reminiscent of the acceleration in mating, in the sense that it is elicited by wasps in a species-specific fashion and is dependent on the LC4 class of VPNs (Fig. 5b).

It is striking that the expression of this gene is also increased by two different stressors, social isolation^32^ and immune challenge^29^. Following immune challenge, the gene is expressed in immune-responsive tissues, including hemocytes and the fat body. We found that after visual contact with wasps it is expressed in the nervous system: in eyes, optic lobe, and other brain tissue. The immune system and the nervous system share a variety of small signaling molecules, including neuropeptides that are produced by and that affect cells of both systems, such as neuropeptide Y^33,34^. There is also extensive communication between the two systems, with the function of each having an impact on the other. We note that two immune peptides, Diptericin B and GNBP-like 3, have recently been found to regulate long term but not short-term memory in *Drosophila*^35^. Further work will be required to determine whether the IBIN micropeptide represents a new link between the two systems.

We have found via genetic analysis that *IBIN* is required for the mating acceleration that is induced by exposure to wasps. Our results now invite investigation of the secreted micropeptide that *IBIN* is predicted to encode. Micropeptides are being found to play roles in a rapidly expanding variety of processes in diverse animals and plants^36^. Further studies are needed to explore the possibility that it binds to a membrane-bound receptor, and to test the hypothesis that it is required for normal activity of neural circuits that control mating behavior. It will be of interest to determine, for example, whether the micropeptide affects the reduction in locomotor activity that is characteristic of female receptivity^37,38^.

Taken together, our results extend the effect of parasitoid wasps to another domain of *Drosophila* behavior: mating. The presence of predators has been found to affect sexual behaviors in other species, including courtship, mate choice, and nest-building in fish^39–41^, calling behavior in tree frogs^42^, and mating behavior in birds^43^. We have examined the effect of exposure to parasitoids, an enormously abundant class of organism, and above all we have studied the effect in *Drosophila*, a species that allows incisive analysis of its underlying molecular, cellular, and circuit basis. Further analysis of the behavioral response we have found in *Drosophila* may have broad implications in the natural world.

## Methods

### Flies

We used our laboratory Canton-S (CS) stock of *Drosophila melanogaster* unless otherwise specified. *Orco*^*1*^ had been backcrossed into a CS genetic background at least five times before using. The *IR8a; Orco* double mutant was from the laboratory of Richard Benton (Univ. Lausanne). Both *Orco*^*1*^ and *IR8a; Orco* were validated via electrophysiology. *nina*^*1*^ (also known as *ninaB*^*P315*^) was from the Bloomington *Drosophila* Stock Center. *D. simulans, D. yakuba, D. biarmipes*, and *D.willistoni* were from the *Drosophila* Species Stock Center. *D. suzukii* was from R. Cowles, Connecticut Agricultural Experiment Station. Additional ethics approval from a board is not required. LC4-SS00315 was from the Janelia Research Campus. *UAS-TNT* was outcrossed to our CS background at least five times before using. All flies were maintained on a standard cornmeal *Drosophila* medium (Archon Scientific Glucose Medium Recipe). Stocks were maintained at 22 °C in 50% relative humidity with a 12:12 light:dark cycle. For stocks maintained in vials, we kept a maximum of 20 females and 20 males together in a vial (9.5 cm height, 2 cm diameter, Archon Scientific) to prevent overcrowding. Stocks kept in bottles (9.5 cm height, 5 cm diameter at base, Archon Scientific) had a maximum of 80 females and 30 males. When flies were near eclosion, parents were removed from the bottles. Newly eclosed flies were transferred to fresh *Drosophila* media (bottles or vials at the same population density) and aged until between 3 and 8 days. Aging the flies on fresh media is important: flies aged on old media (i.e. the media in which they eclosed) appeared nutrient-deprived and laid few eggs.

### Parasitoid wasps

*Leptopilina boulardi* (Lb17), *Leptopilina* *heterotoma*, *Asobara tabida*, and *Trichopria drosophilae* were kindly provided by Todd Schlenke (University of Arizona, Tucson AZ)^44^. In order to culture these wasps, adult *Drosophila* were allowed to lay eggs in vials containing standard *Drosophila* culture medium for 4 days before being replaced by adult wasps (10 females, 10 males), which then attacked the developing fly larvae. Wasps aged 3–7 days post-eclosion were used for all experiments. Fresh wasps were used for all experiments, such that wasps were never reused between experiments. *Muscidifurax zaraptor* was obtained from Rincon-Vitova Insectaries Inc. (Ventura, CA) as pupae and used after eclosion.

### Behavioral assays

To test copulation behavior, a virgin male fly was paired with a virgin female fly, each 3–8-day-old, inside a 35 × 10 mm Petri dish containing *Drosophila* culture medium. The flies had been collected immediately after eclosion and housed in single-sex groups of 10–12 prior to the experiment. The Petri dishes already contained two male and two female wasps (exposed) or no wasps (unexposed), except that in Fig. 1b a single male wasp and a single female wasp were used. Flies and wasps were introduced into the Petri dish using an aspirator. Assays were conducted in a controlled behavioral room set to 25 °C, 50% relative humidity and in room light unless otherwise indicated. We measured copulation latency (time between the introduction of the male and female flies into the mating chamber and the initiation of copulation of each pair). The percentage of assays in which copulation occurred was not consistently greater for either exposed or unexposed flies.

For competition experiments, we placed 10–12 virgin flies (males or females) in a *Drosophila* culture vial (9.5 cm height, 2 cm diameter) with 10 male and 10 female wasps for 2 h. Some flies were labeled, as indicated in some experiments, with UVXPBB UV fluorescent dye (maxmax.com), which had been applied to them by placing them in a vial with the dye and shaking gently, 2 days before the experiment began. We recorded the percentage of competitions won by the exposed fly. To minimize any effect of the label, we averaged the percentage won by the exposed fly in the case when it was fluorescently labeled with the percentage in the case when it was unlabeled; there was no obvious effect of the label. The experimenter did not know which fly was labeled until after the result had been scored. We estimate that copulation occurred in more than 95% of competition experiments, which were allowed to proceed for a maximum of 1 h.

All statistical analysis used non-parametric tests. All experimental values were measured in parallel with age-matched controls. Within an experiment *n* values for experimental and control samples were comparable. We used a power analysis based on initial data (GPower 3.1 (ref. ^45^)) to determine the minimum sample size for copulation latency experiments. Each replicate used different individual flies and wasps. Error bars = SEM.

### Exposure via visual contact

In experiments in which female flies were allowed visual but not physical contact with wasps, 10–12 virgin females were placed in a vial (9.5 cm height, 2 cm diameter) with *Drosophila* culture medium, and the vial was surrounded by six equivalent vials containing culture medium with (exposed) or without (unexposed) 15 male and 15 female parasitic wasps for 2 h.

### Tissue preparation for RNA analysis

Female flies were reared in parallel and kept at 22 °C, 50% humidity. For analysis of whole head RNA, within 1 min after exposure to wasps the flies were flash-frozen in liquid nitrogen and their heads were manually collected over dry ice. Ten heads of 7-day virgin females were collected in each replicate. For analysis of isolated head tissues, fly heads were submerged in phosphate-buffered saline and dissected with forceps under a stereomicroscope. In total, three biological replicates were prepared, each consisting of tissues pooled from five animals.

### RNA extraction

For transcriptome analysis, RT-PCR, and RT-qPCR, tissues were ground in 300 µL RLT lysis buffer (Qiagen) and RNA was extracted by adding 300 µL acid phenol. Samples were heated to 65 °C for 10 min with interruption to vortex the samples. Residual phenol were removed from the aqueous phase with chloroform. Finally, the RNA was precipitated and then dissolved in water. A nanodrop was used to quantify the RNA.

### Transcriptome analysis

RNA quality was determined by a bioanalyzer. RNA library preparation and Illumina HiSeq 2500 sequencing were carried out in the Yale Center for Genome Analysis. The RNA libraries were prepared according to the KAPA mRNA HyperPrep preparation procedure. ~30 million 75-nucleotide long paired-end reads were obtained and processed using the Tuxedo suite^46^. In short, reads were mapped to the *Drosophila melanogaster* genome (Dm6) with TopHat2, default settings, and were quantified used CuffDiff, default settings. In parallel, reads were counted using HTseq^47^ and samples were compared using DESeq2, default settings^48^. Raw reads are accessible at the Genbank SRA database (BioProject accession number PRJNA642090).

### RT-qPCR

cDNA was made from 1 µg of total RNA as a template using EpiScript according to the manufacturer’s protocol (Lucigen). qPCR was carried out with the iTaq Universal SYBR Green (Bio-Rad) system using ~100 ng of cDNA. Target gene expression was normalized to the level of EIF3c transcripts. Primers are provided in Supplementary Table 1.

### Generation of an *IBIN* mutant

A deletion was generated using CRISPR/Cas9 homologous recombination. The deletion lacks nucleotides 97–131 of the annotated *IBIN* transcript; it lacks most of the sequences encoding the predicted mature micropeptide and is predicted to create a frameshift mutation. Guide RNAs (gRNAs) were designed using the flyCRISPR Optimal Target Finder. gRNAs were cloned into pCFD4, a gift from Simon Bullock (Addgene # 49411) directly by PCR using Q5 polymerase (New England Biolabs), pCFD4 as a template. The PCR product, which contains both gRNA templates, was run on a gel and the resulting 600 bp band was gel-purified. In parallel, the original pCFD4 plasmid was digested with *Bbs*I and the linearized plasmid was also gel-purified. The gel-purified products were annealed by Gibson Assembly (NEB) according to the manufacturer’s instructions. The donor vector used for homology-directed repair was pHD-DsRed-attP, a gift from Melissa Harrison & Kate O’Connor-Giles and Jill Wildonger (Addgene #51019), with the *Drosophila* synthetic core promoter and Gal4 sequence from the pBPGUW plasmid, a gift from Gerald Rubin (Addgene #17575), cloned into the 5′ multicloning site, upstream of DsRed^49^. In all, 1 kb length homology arms were PCR-amplified from Canton-S gDNA. Primers are provided in Supplementary Table 1. The homology arms were cloned into the modified pHD-DsRed-attP donor vector by Gibson Assembly. The finalized gRNA expression plasmid and donor vector were injected into embryos with germline-specific expression of Cas9 driven by the *nanos* promoter (y w; nos-Cas9(III-attP2)) by BestGene Inc. (Chino, CA). DsRed-positive alleles were then backcrossed to our control *wCS* line for five generations.

### Statistical analysis

Statistical tests were performed in GraphPad prism (version 6.01).

### Reporting summary

Further information on research design is available in the Nature Research Reporting Summary linked to this article.

## Supplementary information

Supplementary Information

Reporting Summary

## Data Availability

RNA-seq data are available at the Genbank SRA database (accession number SSR13601374; accession number SSR13601375; accession number SSR13601376; accession number SSR13601377). All other data supporting the findings are in the text, figures, and supplementary figures. Source data are provided with this paper.

## Source data

Source Data

## References

[1] Fleury F (2004). Ecological and genetic interactions in *Drosophila*-parasitoids communities: a case study with *D. melanogaster*, *D. simulans*and their common *Leptopilina* parasitoids in south-eastern France. Genetica.

[2] Carton, Y., Bouletreau, M., vanAlphen, J. J. M. & vanLenteren, J. C. in Ashburner, M. et al. *The Genetics and Biology of Drosophila* 347–394 (Academic Press, 1986).

[3] Silvers MJ, Nappi AJ (1986). In vitro study of physiological suppression of supernumerary parasites by the endoparasitic wasp *Leptopilina heterotoma*. J. Parasitol..

[4] Gaston KJ (1991). The magnitude of global insect species richness. Conserv. Biol..

[5] Lemaitre B, Hoffmann J (2007). The host defense of *Drosophila melanogaster*. Annu. Rev. Immunol..

[6] Hwang RY (2007). Nociceptive neurons protect Drosophila larvae from parasitoid wasps. Curr. Biol..

[7] Robertson JL, Tsubouchi A, Tracey WD (2013). Larval defense against attack from parasitoid wasps requires nociceptive neurons. PLoS ONE.

[8] Kacsoh, B. Z., Bozler, J., Ramaswami, M. & Bosco, G. Social communication of predator-induced changes in *Drosophila* behavior and germ line physiology. *Elife* **4**, e07423 (2015). 10.7554/eLife.07423 PMC4456452 25970035

[9] Lefevre T, de Roode JC, Kacsoh BZ, Schlenke TA (2012). Defence strategies against a parasitoid wasp in *Drosophila*: fight or flight?. Biol. Lett..

[10] Kacsoh BZ, Lynch ZR, Mortimer NT, Schlenke TA (2013). Fruit flies medicate offspring after seeing parasites. Science.

[11] Ebrahim SA (2015). *Drosophila* avoids parasitoids by sensing their semiochemicals via a dedicated olfactory circuit. PLoS Biol..

[12] Sturtevant AH (1915). Experiments on sex recognition and the problem of sexual selection in *Drosophila*. J. Anim. Behav..

[13] Greenspan RJ, Ferveur JF (2000). Courtship in *Drosophila*. Annu Rev. Genet..

[14] Fleury F, Gibert P, Ris N, Allemand R (2009). Ecology and life history evolution of frugivorous Drosophila parasitoids. Adv. Parasitol..

[15] Kacsoh BZ, Schlenke TA (2012). High hemocyte load is associated with increased resistance against parasitoids in *Drosophila suzukii*, a relative of *D. melanogaster*. PLoS ONE.

[16] Rota-Stabelli O, Blaxter M, Anfora G (2013). *Drosophila suzukii*. Curr. Biol..

[17] Legner EF, Gerling D (1967). Host-feeding and oviposition on *Musca domestica* by *Spalangia cameroni,**Nasonia vitripennis*, and *Muscidifurax raptor* (Hymenoptera: Pteromalidae) influences their longevity and fecundity. Ann. Entomol. Soc. Am..

[18] Clyne PJ (1999). A novel family of divergent seven-transmembrane proteins: candidate odorant receptors in *Drosophila*. Neuron.

[19] Larsson MC (2004). Or83b encodes a broadly expressed odorant receptor essential for *Drosophila* olfaction. Neuron.

[20] Benton R, Vannice KS, Gomez-Diaz C, Vosshall LB (2009). Variant ionotropic glutamate receptors as chemosensory receptors in Drosophila. Cell.

[21] Abuin L (2011). Functional architecture of olfactory ionotropic glutamate receptors. Neuron.

[22] von Lintig J, Dreher A, Kiefer C, Wernet MF, Vogt K (2001). Analysis of the blind *Drosophila* mutant ninaB identifies the gene encoding the key enzyme for vitamin A formation invivo. Proc. Natl Acad. Sci. USA.

[23] Fischbach K, Dittrich A (2004). The optic lobe of *Drosophila melanogaster*. I. A Golgi analysis of wild-type structure. Cell Tissue Res..

[24] Otsuna H, Ito K (2006). Systematic analysis of the visual projection neurons of *Drosophila melanogaster*. I. Lobula-specific pathways. J. Comp. Neurol..

[25] Wu, M. et al. Visual projection neurons in the *Drosophila* lobula link feature detection to distinct behavioral programs. *Elife* **5**, e21022 (2016). 10.7554/eLife.21022 PMC5293491 28029094

[26] Ache JM (2019). Neural basis for looming size and velocity encoding in the *Drosophila* giant fiber escape pathway. Curr. Biol..

[27] von Reyn CR (2017). Feature integration drives probabilistic behavior in the *Drosophila* escape response. Neuron.

[28] Kacsoh BZ, Bozler J, Bosco G (2018). *Drosophila* species learn dialects through communal living. PLoS Genet..

[29] Valanne S, Salminen TS, Jarvela-Stolting M, Vesala L, Ramet M (2019). Immune-inducible non-coding RNA molecule lincRNA-IBIN connects immunity and metabolism in *Drosophila melanogaster*. PLoS Pathog..

[30] Lefèvre T, Roode JDD, Kacsoh B, Schlenke T (2011). Defence strategies against a parasitoid wasp in *Drosophila*: fight or flight?. Biol. Lett..

[31] Singh ND (2015). Fruit flies diversify their offspring in response to parasite infection. Science.

[32] Agrawal, P., Kao, D., Chung, P. & Looger, L. L. The neuropeptide Drosulfakinin regulates social isolation-induced aggression in *Drosophila*. *J. Exp. Biol.* **223**, jeb207407 (2020). 10.1242/jeb.207407 PMC7033730 31900346

[33] Dantzer R (2018). Neuroimmune interactions: from the brain to the immune system and vice versa. Physiol. Rev..

[34] Kioussis D, Pachnis V (2009). Immune and nervous systems: more than just a superficial similarity?. Immunity.

[35] Barajas-Azpeleta R (2018). Antimicrobial peptides modulate long-term memory. PLoS Genet..

[36] Yeasmin F, Yada T, Akimitsu N (2018). Micropeptides encoded in transcripts previously identified as long noncoding RNAs: a new chapter in transcriptomics and proteomics. Front. Genet.

[37] Bussell JJ, Yapici N, Zhang SX, Dickson BJ, Vosshall LB (2014). Abdominal-B neurons control *Drosophila* virgin female receptivity. Curr. Biol..

[38] Hall JC (1994). The mating of a fly. Science.

[39] Endler J (1987). Endler, J. Predation, light intensity and courtship behaviour in *Poecilia reticulata* (Pisces: Poeciliidae).. Anim. Behav..

[40] Magnhagen C (2004). Reproduction under predation risk in the sand goby, *Pomatoschistus minutes*, and the black goby, *Gobius niger*: the effect of age and longevity. Behav. Ecol. Sociobiol..

[41] Plath M (2019). Predator-induced changes of male and female mating preferences: innate and learned components. Curr. Zool..

[42] Tuttle M, Ryan M (2004). The role of synchronized calling, ambient light, and ambient noise, in anti-bat-predator behavior of a treefrog. Behav. Ecol. Sociobiol..

[43] Santema P, Valcu M, Kempenaers B (2020). Exposure to predator models during the fertile period leads to higher levels of extra-pair paternity in blue tits. J. Anim. Ecol..

[44] Schlenke TA, Morales J, Govind S, Clark AG (2007). Contrasting infection strategies in generalist and specialist wasp parasitoids of *Drosophila melanogaster*. PLoS Pathog..

[45] Faul F, Erdfelder E, Buchner A, Lang AG (2009). Statistical power analyses using G*Power 3.1: tests for correlation and regression analyses. Behav. Res. Methods.

[46] Trapnell C (2012). Differential gene and transcript expression analysis of RNA-seq experiments with TopHat and Cufflinks. Nat. Protoc..

[47] Anders S, Pyl PT, Huber W (2015). HTSeq—a Python framework to work with high-throughput sequencing data. Bioinformatics.

[48] Love MI, Huber W, Anders S (2014). Moderated estimation of fold change and dispersion for RNA-seq data with DESeq2. Genome Biol..

[49] Chahda JS (2019). The molecular and cellular basis of olfactory response to tsetse fly attractants. PLoS Genet.

