# Supplementary material for: Sight of parasitoid wasps accelerates sexual behavior and upregulates a micropeptide gene in Drosophila
Source: Nat Commun. 2021 Apr 27;12:2453. doi: 10.1038/s41467-021-22712-0 (PMC8079388; doi:10.1038/s41467-021-22712-0)
Supplement: Supplementary file 1 — Supplementary Information [file 41467_2021_22712_MOESM1_ESM.pdf]

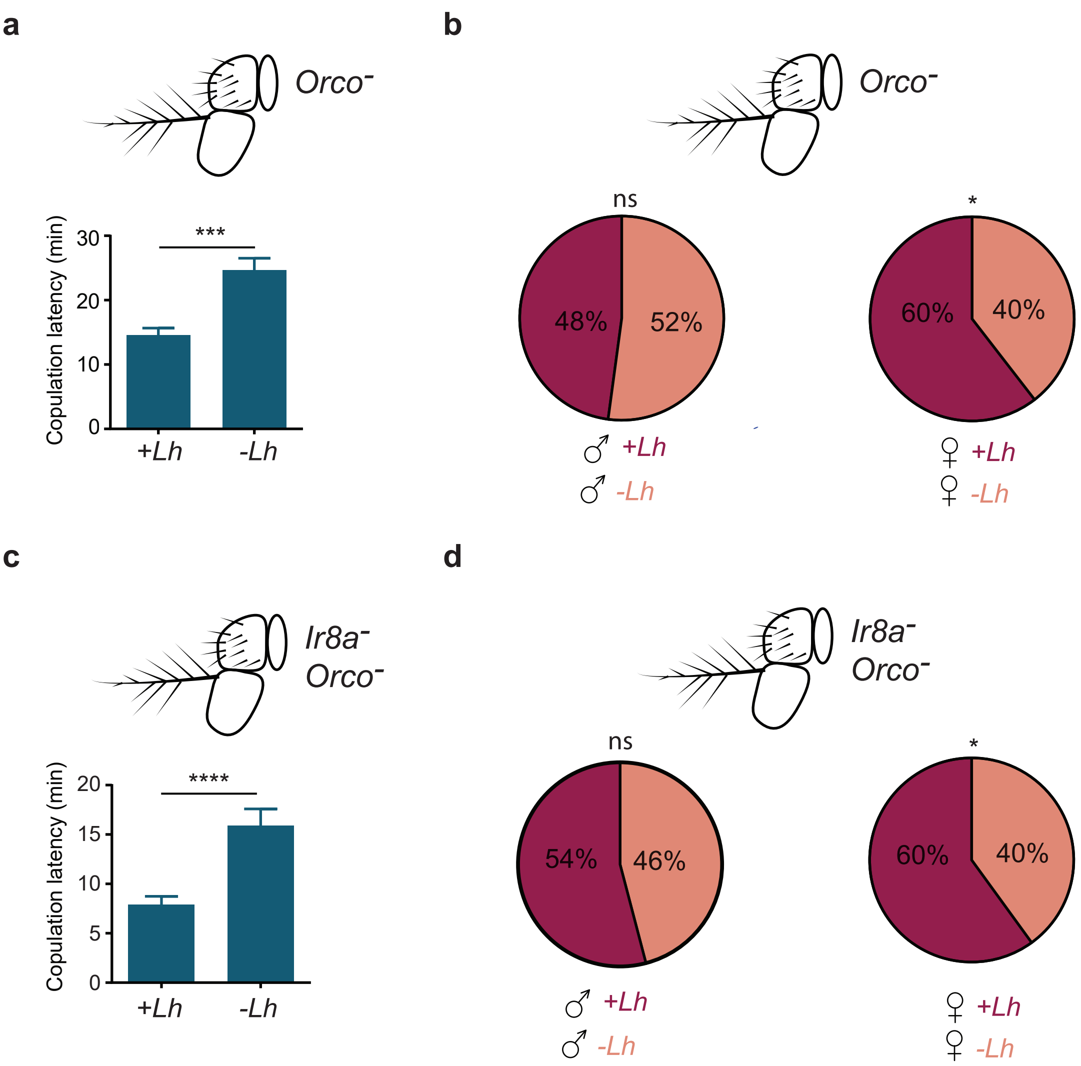

Supplementary Figure 1. The mating acceleration effect depends on visual cues.
a) An *Orco*^{1} mutant is affected by exposure to *L. heterotoma*. p=0.0001, two-tailed Mann-Whitney test, n=71 (exposed), n=70 (unexposed). Error bars=SEM.
b) Left, competition experiments between an exposed and unexposed *Orco*^{1} male, for an *Orco*^{1} female (n=117, p=0.3559, chi-squared test). Right, an exposed and unexposed *Orco*^{1} female are placed with an *Orco*^{1} male (n=124) p=0.0122, chi-squared test.
c) An *Ir8a*; *Orco*^{1} double mutant is affected by exposure to *L. heterotoma*. p<0.0001, two-tailed Mann-Whitney test, n=67 (exposed), n=67 (unexposed). Error bars=SEM.
d) Left, competition experiments between an exposed and unexposed *IR8a*; *Orco*^{1} male, for an *IR8a*; *Orco*^{1} female (n=98, p=0.419, chi-squared test). Right, an exposed and unexposed *IR8a*; *Orco*^{1} female are both placed with an *IR8a*; *Orco*^{1} male p=0.0404, chi-squared test, n=105.

Gene		FPKM			adjusted p-value	
ID	name	control	Lb exp.	log2FC	Cuffdiff	DEseq2
FBgn0265577	**IBIN**	100.7	1809.8	4.2	0.0198	1.9E-10
FBgn0043575	PGRP-SC2	3.4	57.2	4.1	0.0198	4.7E-09
FBgn0038530	AttD	0.7	12.0	4.1	0.0198	1.0E-09
FBgn0262794	CG43175	10.3	145.7	3.8	0.0198	3.9E-13
FBgn0004240	DptA	97.9	1319.7	3.8	0.0198	3.9E-13
FBgn0000278	CecB	2.3	25.5	3.5	0.0198	2.0E-05
FBgn0000279	CecC	4.9	37.0	2.9	0.0198	4.3E-03
FBgn0010388	Dro	1502.0	10326.5	2.8	0.0198	7.5E-09
FBgn0262881	CG43236	114.8	624.3	2.4	0.0198	8.4E-05
FBgn0034407	DptB	106.6	570.8	2.4	0.0198	4.7E-03

Supplementary Figure 2. Differentially expressed genes in the heads of female flies that have been in visual contact with *L. boulardi* for 2h, compared to unexposed flies.
Only significant hits found by both CuffDiff and DESeq2 were considered. Analysis of the *IBIN* gene is highlighted in red. Columns 1-2, gene ID and name; columns 3-4, FPKM (Fragments Per Kilobase of transcript per Million mapped reads) values for control unexposed flies and exposed flies (*Lb* exp.); column 5, log2 fold-change (FC) between exposed and unexposed flies; columns 6-7, p-value adjusted for the false discovery rate calculated with CuffDiff and DESeq2.

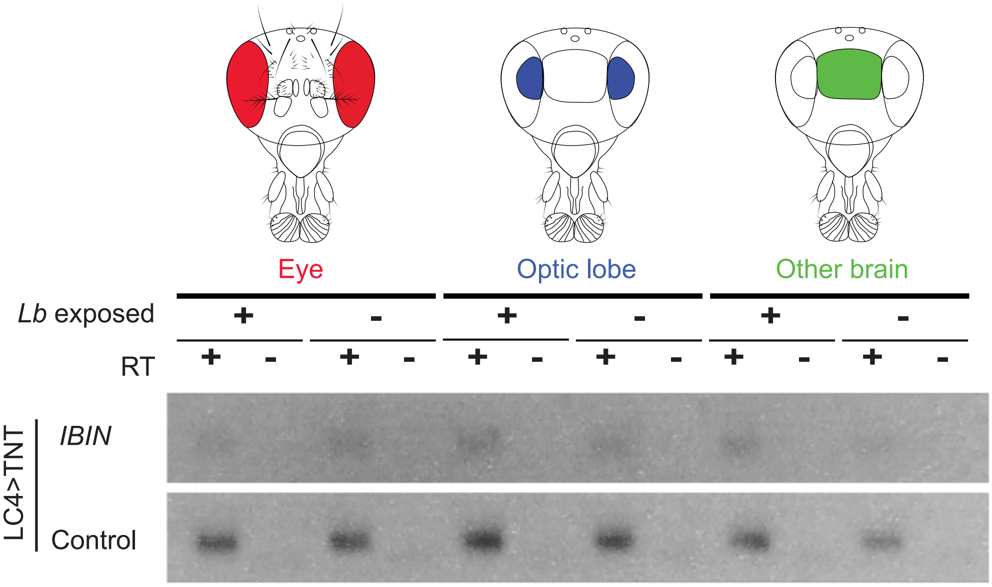

Supplementary Figure 3. RT-PCR amplification from the indicated tissues. RT= reverse transcriptase. The control gene is *eIF3c*. n=2.

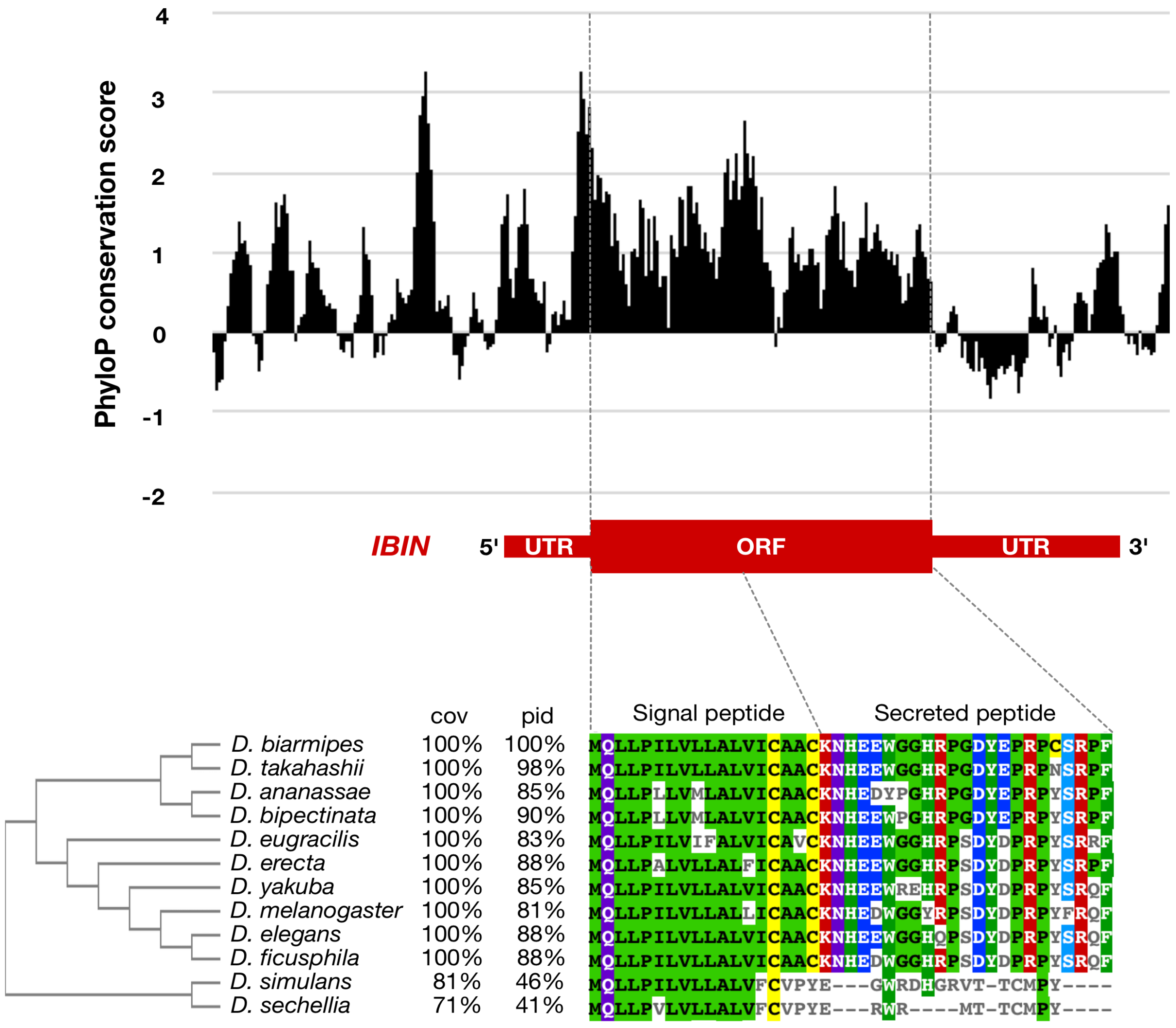

Supplementary Figure 4. ***IBIN* is conserved in other *Drosophila* species.**
The PhyloP conservation score for the locus encoding *IBIN* is based on data from 27 insect species using the University of California Santa Cruz database. The *IBIN* predicted open reading frame (ORF) (average PhyloP score = 1.2) is more conserved than its corresponding untranslated regions (UTRs) (average PhyloP score =0.5). The *IBIN* ORF is more conserved than 15% of all annotated ORFs in the Drosophila genome. Micropeptide alignment was determined using Clustawl Omega[1]and displayed with Mview [2]. Cov = coverage , pid= percentage identity of amino acid sequence relative to the *D.biarmipes* sequence. Conserved amino acids are colored based on their identity. By default, the order and relationship of each species sequence is determined by the alignment.

Supplementary Table 1. List of primers

qPCR primers	IBIN fwd	TGCCAATCCTCGTGCTCCTC
	IBIN rev	CGTGATCATTTAGAACTGCCGGAAATA
	EIF3c fwd	GACTGTGGTCAAGGGTCATGTAG
	EIF3c rev	CTAGCTTCATGGGCTCGGAAAT
primers for the generation of IBIN mutant	guideRNA fwd	TATATAGGAAAGATATCCGGGTGAACTTCG ACCAAGTGACTACGATCCCGTTTTAGAGCTAGAAATAGCAAG
	guideRNA rev	ATTTTAACTTGCTATTTCTAGCTCTAAAACCGAGGACTGGGGAGGATACCGACGTTAAATTGAAAATAGGTC
	homology arm 1 fwd	CGAAAGACTGGGCCTTTCGCAGAGCTTCACTCCACAAATGAAC
	homology arm 1 rev	AGCTGAACGATCGTGGTTCTTGCAGGCG
	homology arm 2 fwd	CGAGTTCAAGCCCAGGCCATATTTCCGG
	homology arm 2 rev	AGAATAAATTTTGTGTCGCCCTGGTGGAGTTCCTCACC

Supplementary References

1. Sievers F*, et al.* Fast, scalable generation of high-quality protein multiple sequence alignments using Clustal Omega. *Mol Syst Biol* **7**, 539 (2011).

2. Brown NP, Leroy C, Sander C. MView: a web-compatible database search or multiple alignment viewer. *Bioinformatics* **14**, 380-381 (1998).
