# Supplementary material for: Sight of parasitoid wasps accelerates sexual behavior and upregulates a micropeptide gene in Drosophila
Source: Nat Commun. 2021 Apr 27;12:2453. doi: 10.1038/s41467-021-22712-0 (PMC8079388; doi:10.1038/s41467-021-22712-0)
Supplement: Supplementary file 2 — Reporting Summary [file 41467_2021_22712_MOESM2_ESM.pdf]

Corresponding author(s): John Carlson

Last updated by author(s): 2021/03/16

Reporting Summary

Nature Research wishes to improve the reproducibility of the work that we publish. This form provides structure for consistency and transparency in reporting. For further information on Nature Research policies, see our Editorial Policies and the Editorial Policy Checklist.

Statistics

For all statistical analyses, confirm that the following items are present in the figure legend, table legend, main text, or Methods section.

n/a	Confirmed	
☐	☒	The exact sample size (*n*) for each experimental group/condition, given as a discrete number and unit of measurement
☐	☒	A statement on whether measurements were taken from distinct samples or whether the same sample was measured repeatedly
☐	☒	The statistical test(s) used AND whether they are one- or two-sided<br>*Only common tests should be described solely by name; describe more complex techniques in the Methods section.*
☒	☐	A description of all covariates tested
☒	☐	A description of any assumptions or corrections, such as tests of normality and adjustment for multiple comparisons
☐	☒	A full description of the statistical parameters including central tendency (e.g. means) or other basic estimates (e.g. regression coefficient) AND variation (e.g. standard deviation) or associated estimates of uncertainty (e.g. confidence intervals)
☐	☒	For null hypothesis testing, the test statistic (e.g. F, t, r) with confidence intervals, effect sizes, degrees of freedom and P value noted<br>*Give P values as exact values whenever suitable.*
☒	☐	For Bayesian analysis, information on the choice of priors and Markov chain Monte Carlo settings
☒	☐	For hierarchical and complex designs, identification of the appropriate level for tests and full reporting of outcomes
☒	☐	Estimates of effect sizes (e.g. Cohen's *d*, Pearson's *r*), indicating how they were calculated

Our web collection on statistics for biologists contains articles on many of the points above.

Software and code

Policy information about availability of computer code

Data collection	No software was used
Data analysis	GraphPad Prism (version 8.0.0) ; HTseq (version 0.12.4); DESeq2 (Version 3.12); Tuxedo suite: TopHat (version 2.1.1) and CuffDiff (version 2.2.1).

For manuscripts utilizing custom algorithms or software that are central to the research but not yet described in published literature, software must be made available to editors and reviewers. We strongly encourage code deposition in a community repository (e.g. GitHub). See the Nature Research guidelines for submitting code & software for further information.

Data

Policy information about availability of data

All manuscripts must include a data availability statement. This statement should provide the following information, where applicable:

- Accession codes, unique identifiers, or web links for publicly available datasets
- A list of figures that have associated raw data
- A description of any restrictions on data availability

RNA-seq data are available at the Genbank SRA database (accession number SSR13601374, https://www.ncbi.nlm.nih.gov/sra/?term=SRR13601374; accession number SSR13601375, https://www.ncbi.nlm.nih.gov/sra/?term=SRR13601375; accession number SSR13601376, https://www.ncbi.nlm.nih.gov/sra/?term=SRR13601376; accession number SSR13601377, https://www.ncbi.nlm.nih.gov/sra/?term=SRR13601377). All other data supporting the findings are in the text, figures, and supplementary figures. Source data are provided with this paper.

Field-specific reporting

Please select the one below that is the best fit for your research. If you are not sure, read the appropriate sections before making your selection.

☒ Life sciences ☐ Behavioural & social sciences ☐ Ecological, evolutionary & environmental sciences

For a reference copy of the document with all sections, see nature.com/documents/nr-reporting-summary-flat.pdf

Life sciences study design

All studies must disclose on these points even when the disclosure is negative.

Sample size	We used a power analysis based on initial data to determine the minimum sample size.
Data exclusions	No data were excluded from the analyses.
Replication	All attempts at replication were successful. The n of independent experiments is indicated in the figure legends.
Randomization	The experimental groups were allocated based on genotypes. Appropriate control groups were assayed in parallel. Randomization was irrelevant for the design of this study.
Blinding	The experimenter did not know which fly was labeled until after the result had been scored.

Reporting for specific materials, systems and methods

We require information from authors about some types of materials, experimental systems and methods used in many studies. Here, indicate whether each material, system or method listed is relevant to your study. If you are not sure if a list item applies to your research, read the appropriate section before selecting a response.

Materials & experimental systems

n/a	Involved in the study	
☒	☐	Antibodies
☒	☐	Eukaryotic cell lines
☒	☐	Palaeontology and archaeology
☐	☒	Animals and other organisms
☒	☐	Human research participants
☒	☐	Clinical data
☒	☐	Dual use research of concern

Methods

n/a	Involved in the study	
☒	☐	ChIP-seq
☒	☐	Flow cytometry
☒	☐	MRI-based neuroimaging

Animals and other organisms

Policy information about studies involving animals; ARRIVE guidelines recommended for reporting animal research

Laboratory animals	Drosophila melanogaster, D. simulans, D. yakuba, D. biarmipes, D. willistoni, D. suzukii, Leptopilina boulardi, L. heterotoma, Asobara tabida, Trichopria drosophilae, Muscidifurax zaraptor, transgenic Drosophila melanogaster strain UAS-TNT, transgenic Drosophila melanogaster strain LC4-SS00315. 3-8 day old animals of both sexes were used.
Wild animals	The study did not involve wild animals.
Field-collected samples	The study did not involve samples collected from the field.
Ethics oversight	Details of animal handling are included in the method section. Stocks were maintained at 22C in 50% relative humidity with a 12:12 light: dark cycle.

Note that full information on the approval of the study protocol must also be provided in the manuscript.
