# Supplementary figures and images for: Sight of parasitoid wasps accelerates sexual behavior and upregulates a micropeptide gene in Drosophila
Source: Nat Commun. 2021 Apr 27;12:2453. doi: 10.1038/s41467-021-22712-0 (PMC8079388; doi:10.1038/s41467-021-22712-0)

Figure 5c


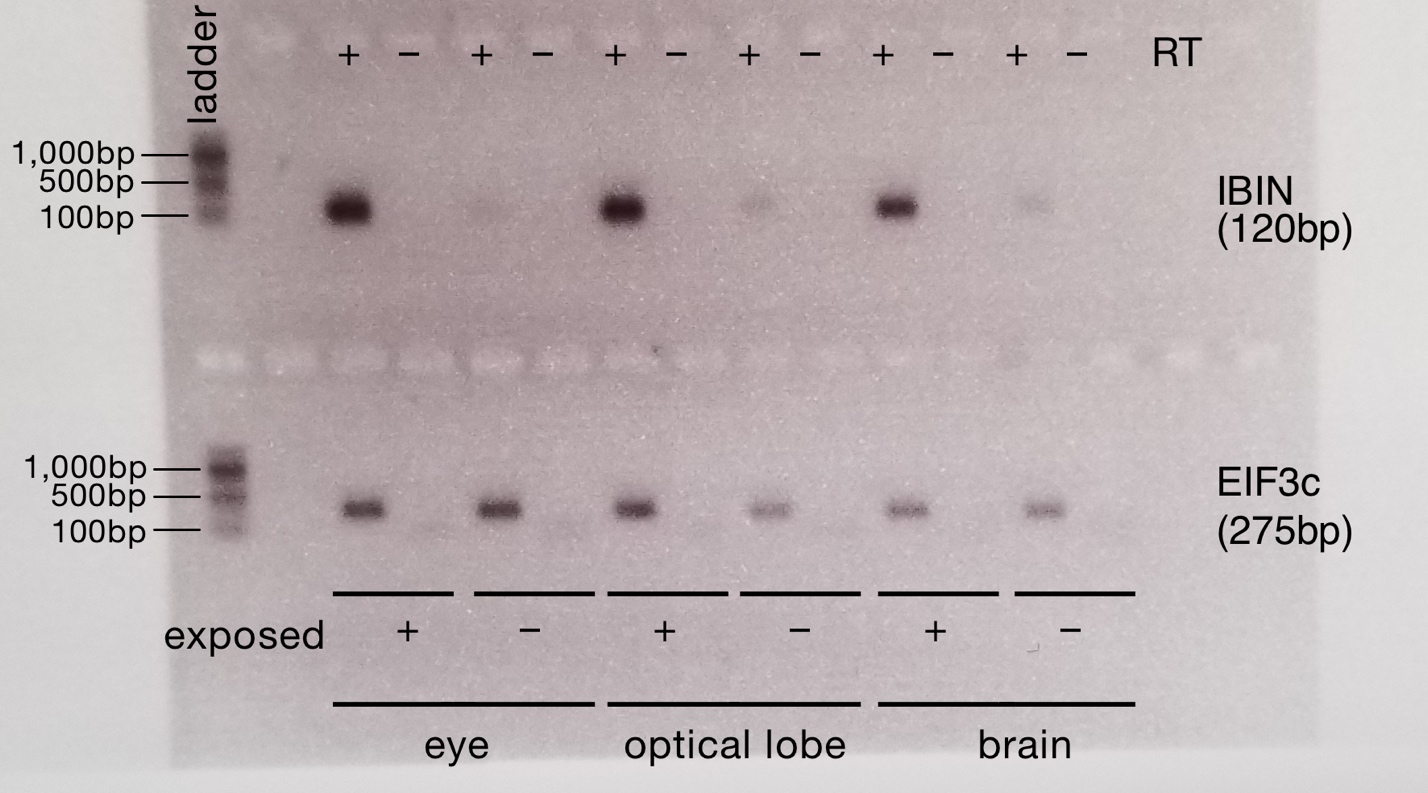


Figure S3


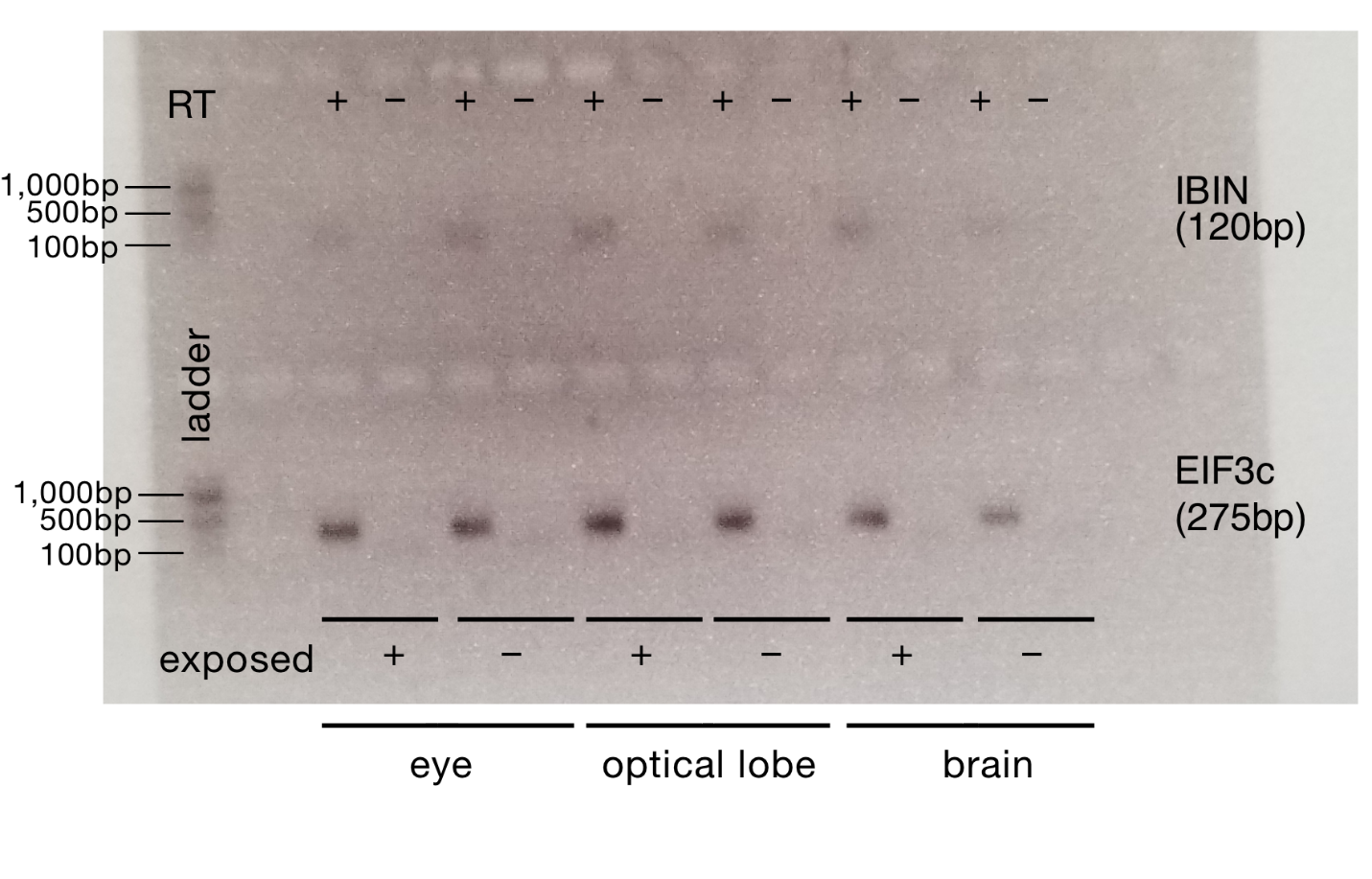

Supplement: Supplementary file 3 — Source Data [file 41467_2021_22712_MOESM3_ESM.zip › Full_gel_images.docx]
